# Aortopathies: From Etiology to the Role of Arterial Stiffness

**DOI:** 10.3390/jcm12123949

**Published:** 2023-06-09

**Authors:** Giovanni Battista Bonfioli, Luca Rodella, Roberta Rosati, Alberto Carrozza, Marco Metra, Enrico Vizzardi

**Affiliations:** Department of Medical and Surgical Specialties, Radiological Sciences and Public Health, ASST Spedali Civili di Brescia, Cardiology University of Brescia, 25123 Brescia, Italy; g.bonfioli@unibs.it (G.B.B.); l.rodella004@unibs.it (L.R.); rosati.roberta@hotmail.it (R.R.); carrozza.alberto@unibs.it (A.C.); marco.metra@unibs.it (M.M.)

**Keywords:** aorta, aortopathies, genetics, heritable connective tissue disorders, arterial stiffness, pulse wave velocity

## Abstract

The aorta and aortic wall have a complex biological system of structural, biochemical, biomolecular, and hemodynamic elements. Arterial stiffness could be considered a manifestation of wall structural and functional variations, and it has been revealed to have a strong connection with aortopathies and be a predictor of cardiovascular risk, especially in patients affected by hypertension, diabetes mellitus, and nephropathy. Stiffness affects the function of different organs, especially the brain, kidneys, and heart, promoting remodeling of small arteries and endothelial dysfunction. This parameter could be easily evaluated using different methods, but pulse-wave velocity (PWV), the speed of transmission of arterial pressure waves, is considered the gold standard for a good and precise assessment. An increased PWV value indicates an elevated level of aortic stiffness because of the decline in elastin synthesis and activation of proteolysis and the increase in fibrosis that contributes to parietal rigidity. Higher values of PWV could also be found in some genetic diseases, such as Marfan syndrome (MFS) or Loeys-Dietz syndrome (LDS). Aortic stiffness has emerged as a major new cardiovascular disease (CVD) risk factor, and its evaluation using PWV could be very useful to identify patients with a high cardiovascular risk, giving some important prognostic information but also being used to value the benefits of therapeutic strategies.

## 1. Aorta: Embryogenesis and Anatomy

Inside the aorta there is a complex biological system of different structural, biochemical, biomolecular, and hemodynamic elements, all coexisting in order to maintain homeostasis [1]. Moreover, there is embryological and morpho-functional heterogeneity between the different aortic segments, which explains the different localization of aortic diseases.

### 1.1. Structure of the Aortic Wall

The aortic wall is structurally divided into three tunicae (intima, media, and adventitia). Tunica media constitutes 90% of the parietal thickness and has a structure capable of reabsorbing the hemodynamic forces exerted by cardiac systole. The structural unit of the tunica media is the muscular-elastic lamella (lamellar unit), which is composed of vascular smooth muscle cells (VSMCs) placed between two layers of elastin fibres, which contain microfibrils and proteoglycans that form the extracellular matrix (ECM). The lamellar unit has both tensile strength and elastic recoil properties, allowing the aorta to withstand high pressures and return to its initial diameter during diastole [2,3]. Tunica intima interfaces with circulating blood and is composed of a single continuous layer of endothelial cells placed in loose connective tissue with scattered rare smooth muscle cells. Tunica adventitia has an immune surveillance function and contains fibroblasts, dendritic reticular cells, collagen fibres, and scarce elastic lamellae.

Aortic function is provided by the interaction between cellular elements (endothelium, muscle cells, and fibroblasts) and ECM proteins. VSMCs have both contractile and secretory properties and can ensure the synthesis of various components of the extracellular matrix (collagen, elastin, fibrillin, or fibulin) in response to mechanical or biochemical stimuli, such as transforming growth factor-β1 (TGF-β1). The capability to transform mechanical stimuli into biological responses (mechano-transduction) depends on the interaction between the cytoskeleton of myocells and the ECM [4]. Apoptosis of VSMCs and a decrease in VSMC density have been identified as hallmarks of aortic tissue degeneration that contribute to aneurysm formation [5,6].

The components of the extracellular matrix determine the static properties of the aortic wall. The most represented types of collagens are types I and III, which provide resistance to tensile forces and act on cell adhesion and proliferation through binding to cell surface receptors [2]. Elastin confers elastic distension and retraction capabilities and plays an active role in promoting the proliferation and migration of smooth muscle cells. The fibrillar matrix also includes the complex of microfibrils associated with elastin, whose main components are represented by fibrillin 1 and 2 and fibulin, proteins that play a role in the structural support of elastin and the formation of lamellar units [7].

Mutations involving ECM components (e.g., mutations in genes FBN-1, COL3A1, COL1A1, COL5A1), the TGF-β1 pathway (e.g., TGFBR1 and 2, SLC2A10), and cytoskeletal proteins in the VSMCs (e.g., ATCA2 or MYH11) lead to heredo-familial or sporadic aortopathies [2].

### 1.2. Embryological, Structural, and Mechanical Heterogeneity

There is marked heterogeneity between the thoracic and abdominal parts of the aortic wall, which is already present during embryogenesis. The ascending aorta, the aortic valve, and the ductus arteriosus originate from neural crest cells, while the abdominal aorta originates from mesodermal-derived cells, leading to different responses to various cytokines and growth factors [8,9]. These two segments also have an important morpho-functional heterogeneity: the middle layer has 55–60 lamellar units in the ascending aorta, while the abdominal aorta has only 28–32 units. This different organization makes the thoracic aorta more elastic and distensible than the abdominal one. Fewer lamellar units make the wall tension per unit in the abdominal aorta higher, thus influencing VSMCs transcriptional activity and therefore matrix structure. Furthermore, the entire abdominal tunica media is not vascularized, and the delivery of oxygen occurs exclusively by diffusion from the lumen, whereas the thoracic media contain vasa vasorum, which originate by adventitia [9,10].

### 1.3. Biochemical and Molecular Regulatory Systems

The TGF-β1 system has a central role both in cardiac and vascular morphology as well as in ECM homeostasis. TGF-β1 contributes both to extracellular matrix synthesis through stimulation of collagen and elastin production and matrix degradation, assuming a major role in the development of thoracic aneurysms [11]. Proteolysis is regulated by matrix metalloproteases (MMPs) and their inhibitors (tissue inhibitors of metalloproteinases—TIMPs); the balance of their actions determines the actual composition of the extracellular matrix of the aortic wall. MMPs are enzymes that degrade the extracellular matrix, thus contributing to parietal remodelling and playing an important pathogenetic role in the development of aneurysms. They are produced by different types of cells (including VSMCs) as a response to different stimuli, such as inflammation; this could explain how the inflammatory response, regulated by cytokines, may be involved in aortic parietal remodelling and in the development of various aortic diseases [12]. Different expression of MMPs between the thoracic and abdominal aortas can account for the different incidence of aneurysms [9].

## 2. Causes of Aortic Disease

There are many different mechanisms behind aortopathies. The main causes of aortic disease can be considered: (i) Atherosclerosis, a multifactorial disease that hits muscular and elastic arteries. The plaque is composed of a fibrous cap and lipids, collagen, calcium, and inflammatory infiltrates. Its development is associated with the media’s thinning and an adventitia’s inflammatory reaction that can alter the periaortic tissue; (ii) malformative pathology of the aorta, often associated with congenital and/or valve diseases. A classic example is the bicuspid aortic valve (BAV); (iii) inflammatory diseases with either infectious or non-infectious causes. Nowadays, autoimmune diseases, such as giant cell arteritis and Takayasu arteritis, prevail due to infections that can be both bacterial and fungal; (iv) degenerative disease, either genetic or mediated by other mechanisms such as hypertension or aging; (v) trauma; (vi) cancer, both primitive (sarcoma) or secondary to infiltration of the aortic wall by neoplasia located in the mediastinum; and (vii) other causes, such as cocaine abuse, weightlifting, or severe physical exertion [1,13,14].

This classification is primarily focused on a macroscopic evaluation of the vessel, derived from autopsy studies. In the last two decades, scientific advances have allowed us to enrich this first classification with histopathological studies, creating a bridging analysis between biomolecular processes and clinical findings. Based on this new approach, a new kind of classification is possible, differentiating between inflammatory and non-inflammatory aortopathies.

Inflammatory infiltrates are typical findings in atherosclerosis, aortitis, and chronic periaortitis. Atherosclerotic plaques have multiple precipitating etiological factors, including age, lipid metabolism, and vascular cell activation, and are associated with inflammation, which can contribute to complications such as aneurysm formation and surface disruption with thrombus formation. Aortitis and chronic periaortitis can be due to both infectious and non-infectious conditions, such as autoimmune diseases [15].

Non-inflammatory aortopathies are related to degenerative changes of the media, specifically impacting the lamellar unit, with the possible development of aneurysms. Medial degeneration is the primary pathologic substrate both in heritable connective tissue diseases of the aorta and in some nongenetic causes of aortic aneurysms such as hypertension, cocaine abuse, weightlifting/severe physical exertion, and pregnancy [16].

## 3. Aortopathies and Genetics

While abdominal aortic aneurysms (AAAs) are typically degenerative disorders associated with traditional atherosclerotic factors such as advanced age, smoking, hypertension, and hypercholesterolemia, thoracic aortic aneurysms (TAAs) are more likely associated with genetic background, including both syndromic aortopathies, mainly heritable connective tissue disorders (HCTDs), and non-syndromic aortopathies [17,18]. The definition of heritable connective tissue disorders (HCTDs) includes Marfan syndrome (MFS), Ehlers–Danlos syndrome (EDS), Loeys–Dietz syndrome (LDS), imperfect osteogenesis, and arterial tortuosity syndrome (ATS) (Table 1).

The interest in aortopathies’ genetics began after discovering the association between Marfan syndrome and the fibrillin gene’s mutations [19]. Marfan syndrome is an autosomal dominant connective tissue disease with an estimated incidence of 1 in 5000 individuals. The cardiovascular, ocular, and skeletal systems are the main ones affected [20,21]. Type 1 MFS (or classic type) is associated with the FBN-1 mutation, representing 90% of all cases, and has a wider spectrum of manifestations, including ectopia lentis, which, together with ascending aorta dilatation or dissection, is a major criteria in MFS’ diagnosis, while symptoms of type 2 MFS, which can be associated both with FBN-1 or transforming growth factor receptor β 1 or 2 (TGFBR1 or TGFBR2) mutations, affect only the skeletal and cardiovascular systems. About one quarter of affected individuals have no family history, accounting for de novo mutations [22]. In MFS, extracellular matrix (ECM) is abnormal, and the continuous stress mediated by the strength of the blood flow ejected from the heart activates the TGF-βsignaling pathway to restore it. However, the excessive TGF-β signaling causes ECM degradation, cells apoptosis and an inflammatory state, leading to aneurysm formation or dissection. Because of the continual force derived from the left ventricular cyclic torsion, aortic root dilatation is the most common cardiovascular manifestation of MFS, followed by ascending aortic and thoracic aortic aneurysms (TAA), which can precipitate aortic regurgitation. Mitral valve prolapse (MVP), is the most common valvular abnormality, affecting 35–100% of patients. Tricuspid regurgitation (TR), pulmonary artery (PA) dilatation, ventricular arrhythmia, and dilated cardiomyopathy may also occur [22,23,24]. According to Ghent nosology, aortic root aneurysm/dissection end ectopia lentis combined are sufficient to make the diagnosis of MFS; however, a “systemic score” is also included in the definition, as the skeleton, dura, skin, and lungs may be affected too [25]. Recently, Ghent nosology was revised in order to be more accurate in differentiating between MFS and other syndromes with overlapping manifestations. MASS syndrome, in particular, is also caused by an FBN-1 mutation. MASS stands for mitral valve prolapse, aortic dilation, generally mild and nonprogressive, and nonspecific skin and skeletal marfanoid features. Compared to MFS, MASS syndrome is not associated with ectopia lentis. However, it is not easy to distinguish one from the other, especially during childhood, and therefore a diagnosis can be established only for individuals aged 20 or older [26].

Loeys–Dietz Syndrome is an aggressive genetic condition that predisposes an individual to the development of life-threatening aortic aneurysms [27]. It is characterized by the diagnostic triad of generalized arterial tortuosity with rapidly progressive aortic aneurysms, hypertelorism, and a bifid/broad uvula or cleft palate. LDS patients do not have ectopia lentis, but blue sclera can be an important clue. In a revised nosology for the diagnosis of LDS, patients are classified based on the presence of different mutated genes: TGFBR1 (LDS-1), TGFBR2 (LDS 2), SMAD3 (LDS 3), and TGFB2 (LDS 4) [28,29,30].

Ehlers–Danlos syndromes (EDS) are characterized by a variable degree of skin hyperextensibility, joint hypermobility, and tissue fragility. The last version of the EDS classification distinguishes 13 subtypes and 19 different causal genes mainly involved in collagen and extracellular matrix synthesis and maintenance [31]. The most common variants are hypermobile EDS (hEDS, 1:3100–5000), classical EDS (cEDS, 1:20,000–40,000) and vascular EDS (vEDS, 1:100,000–200,000). (www.ehlers-danlos.com accessed on 24 April 2023). The most frequently mutated gene in vEDS is COL3A1, which encodes for collagen type III, followed by COL1A1, which is less common and encodes for collagen type I. Vascular EDS is characterized by arterial fragility with aneurysm development, dissection, and rupture, and in general by organ fragility, extensive bruising, and pneumothorax. A family history of the disorder should lead to targeted diagnostic studies, especially in patients with a familiar history of arterial dissection in individuals less than 40 years of age, unexplained sigmoid colon rupture, or spontaneous pneumothorax. To reach a correct diagnosis, it is important to identify mutations in the causative genes. Modulation of lifestyle to minimize injury and an aggressive anti-hypertensive strategy are the most important recommendations [31,32].

Osteogenesis imperfecta results from deletions, insertions, or exon slice errors in the genes encoding type I collagen pro-α1 and pro-α2 chains, but mutations remain unknown in most cases. The diagnosis is made by clinical assessments of symptoms, which include bone fragility, defective skeletal development, smaller stature, blue sclerae, and hyperextensible ligaments. Most cases are associated with pathogenic variants in COL1A1 and COL1A2, while about 25% of cases are associated with other genes that function within the collagen biosynthesis pathway or are involved in osteoblast differentiation and bone mineralization. Altered type I collagen synthesis can affect the aortic wall and cardiac valves biomechanical properties, leading, in rare cases, to aortic dissection. The main cardiovascular features are aortic root dilatation and mostly left-sided valve pathologies [33,34,35].

Arterial tortuosity syndrome (ATS) is characterized by widespread elongation and tortuosity of the aorta and mid-sized arteries, as well as focal stenosis of segments of the pulmonary arteries and/or aorta. Other features include hyperxtensible skin, joint hypermobility, or hernia (both diaphragmatic or inguinal), and skeletal malformations such as pectus excavatum or carinatum, arachnodactyly, scoliosis, knee/elbow contractures, and camptodactyly. The cardiovascular system is the major source of morbidity and mortality, with an increased risk of aneurysm formation and dissection involving both the aortic root and the whole arterial tree at any age. The mutated gene is solute carrier family 2 member 10 (SLC2A10), encoding for GLUT10, and the disease has a recessive autosomal inheritance. Aortic root aneurysms are found in 16% of ATS’ patients; they can evolve aggressively during early childhood or be slowly progressive during adolescence or adulthood. Pulmonary artery and aortic stenosis are also frequent findings (57% and 24%, respectively) [36].

Differently from aortopathies in the HCTD contest, non-syndromic aneurysms and/or dissections are not associated with skeletal manifestation, mitral valve prolapse, or other vascular defects such as arterial tortuosity but can be defined as isolated. We can cluster non-syndromic aneurysms, primarily thoracic aortic aneurysms, as familial (fTAA) or sporadic (sTAA), based on the presence of affected family members. Familial aneurysms present earlier in life and have a higher annual growth rate. Modern genome sequencing technologies have identified several genetic lesions associated with the development of TAA that show familial aggregation, such as mutations in ACTA2 (the most common one, accounting for 10–15% of fTAA), but also in MYH11, MYLK, PRKG1, and FBN1 [37,38].

Non-syndromic aneurysms are often associated with the bicuspid aortic valve (BAV). BAV is the most common congenital heart defect, with a prevalence estimated between 0.5 and 2%. Patients can present with a severe complication, detected in utero, or be completely asymptomatic even in old age. The main complications of BAV are valve stenosis, endocarditis, aortic aneurysms, and/or dissection [39,40]. Even if the genetics of BAV are mostly unknown, some studies have shown a correlation with mutations in genes such as NOTCH1 and GATA5 [41]. BAV can be isolated or part of more complex syndromes, such as Turner syndrome. Furthermore, an association between Marfan syndrome, FBN mutations, and BAV has been reported [42].

It is important to identify possible syndromic and familial aortopathies by assessing both extravascular symptoms and family history. Genetic testing can be useful to confirm suspicion but cannot be used to differentiate between different diseases with overlapping manifestations, because mutations of the same gene can be related to different syndromes (for example, mutations of FBN-1 in MFS and MASS).

## 4. Aortic Wall Physiopathology and Pulse Wave Velocity (PWV)

The biomechanical properties of the aortic wall determine the pathophysiology of many cardiovascular diseases. Arterial stiffness is a manifestation of wall structural and functional variations and has been demonstrated to be an independent predictive value for cardiovascular events in patients with hypertension, diabetes mellitus, and end-stage renal disease. The functional evaluation of this parameter is based on some indices (Table 2) that can be calculated using different methods and techniques (applanation tonometry, echocardiography, and magnetic resonance imaging), which have different sensitivity according to the patient’s age and to the affected aortic segment [43,44,45].

**Figure 1:**
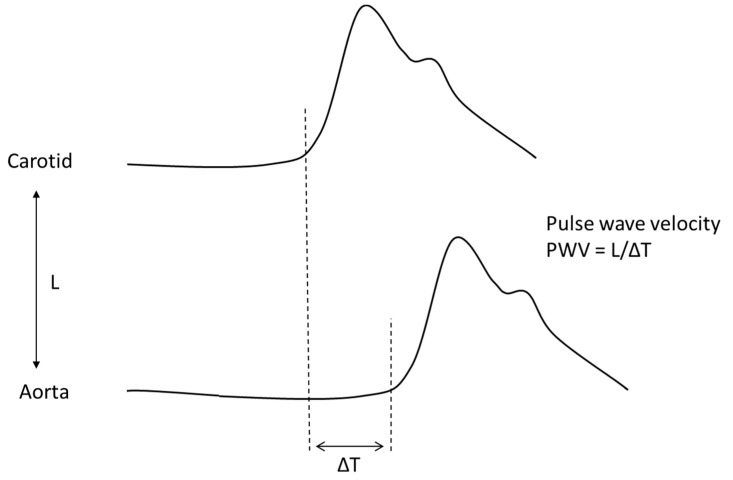
Non-invasive determination of pulse wave velocity (PWV) between the carotid artery and the terminal aorta. PWV is determined by the ratio between the distance from the carotid to the terminal aorta (L) and the differential time between the arrival of the pulse wave in the two districts (ΔT).

The clinical and prognostic significance of functional indices of the aorta has mostly been studied in the context of the stratification of cardiovascular risk. The speed of transmission of arterial pressure waves (pulse wave velocity, PWV) in the carotid-femoral joint (cfPWV), considered the gold standard for arterial stiffness assessment, is an independent predictor of coronary artery disease and stroke in healthy subjects (Figure 1). PWV is also a predictor of mortality in the general population and in diabetic patients in particular [46,47,48]. Reference values for PWV are available in healthy populations and in patients at increased cardiovascular (CV) risk. A PWV > 10 m/s reflects a significant alteration of aortic function [49].

Large artery stiffening (LAS) precedes isolated systolic hypertension and establishes a cycle of hemodynamic stress leading to endothelial disfunction, which includes inward remodeling of small arteries, which increases resistance and blood pressure. Arterial stiffness can reflect vascular aging, be an early marker of vascular disfunction, and therefore be a useful therapeutic target in cardiovascular prevention [50,51,52].

Stiffness results in an increase in left ventricular systolic load and consequently an increase in myocardial oxygen demand, possibly leading to cardiac hypertrophy, fibrosis, and/or heart failure (HF). The excessive pressure pulsatility and the loss of aortic compliance can also lead to diastolic disfunction and, consequently, a reduction in coronary perfusion, aggravating the myocardial oxygen supply/demand ratio. The heart is not the only organ affected by arterial stiffness. Stiffness can lead to an increase in pulsatile loads in the microvascular bed, and in particular, the brain and kidneys can suffer the consequences, as they both require high blood flow and low resistance heamodynamics. A rise in cfPWV was associated both with chronic kidney disease (CKD) and with cognitive decline and incident dementia. The link between cfPWV and the progression of CKD and albuminuria has been demonstrated in particular in type-1 diabetes (T1D) patients, where it was also found to have a direct correlation with cardiovascular events and all-cause mortality. Therefore, the assessment of arterial stiffness in T1D can be useful to monitor the progression of the pathology [50,53,54].

**Figure 2:**
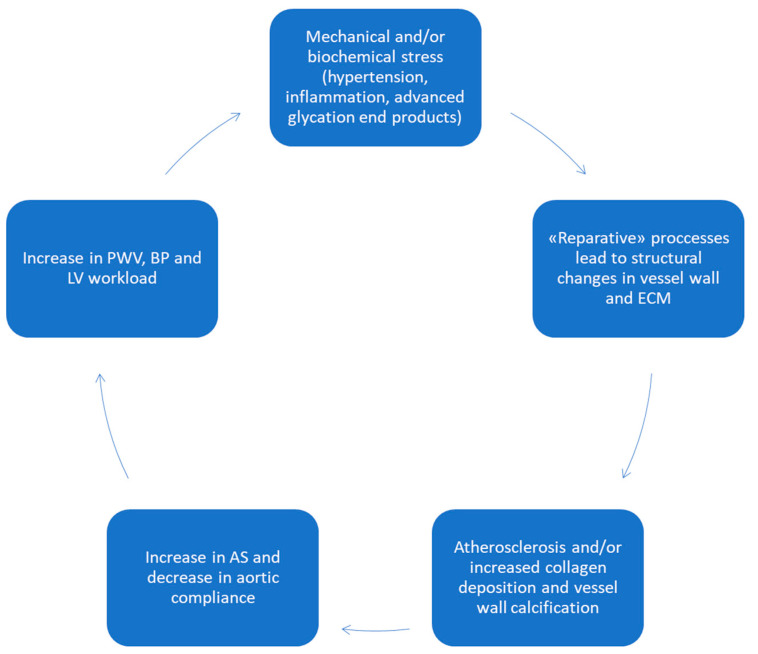
Insults leading to structural changes in the aorta and its functioning. Legend: AS, arterial stiffness; BP, blood pressure; ECM, extracellular matrix; LV, left ventricular; PWV, pulse wave velocity.

It seems that stiffening of the aortic wall begins in the third decade of age because of thinning and fragmentation of the induced elastic fibres under pulsatile stress. The physiopatological mechanisms consist of a decline in elastin synthesis and activation of proteolysis [55,56]. In addition, the increase in fibrosis contributes to parietal rigidity. Factors that contribute to aortic stiffness are complex and incompletely elucidated, but they include the remodeling and breakdown of long-lived elastic fibres and the progressive deposition of much stiffer collagen fibres in the aortic wall. Since the pool of elastin is relatively, fixed from early childhood onward, aortic remodeling thins the pool of elastic fibres and, by the law of Laplace, places the fibres under higher tension. The combination of thinning and enhanced tension increases elastic fibres stress, resulting in a greater engagement of collagen [57]. Fragmentation and loss of elastic fibres amplify the load of the remaining fibres and cause augmented deposition and crosslinking of collagen, contributing to the stiffening of the aortic wall. Elastic fragmentation products, as well as inflammation, may activate signalling pathways that lead to VSMCs dedifferentiation from a contractile to a secretory phenotype (osteogenic differentiation), promoting arterial stiffening and vascular calcification [50,58]. All these modifications contribute to vascular aging, which can be accelerated by many risk factors such as hypertension, diabetes, and individual behaviour (e.g., smoking, alcohol intake, and physical activity). We can refer to arterial stiffening resulting from vascular aging as arteriosclerosis, which differs from atherosclerosis, which is a process beginning with a local lesion in the intima and continuing with lipid deposition and consequent inflammation. The pathological expression of arteriosclerosis is dilatation, while that of atherosclerosis is stenosis. Arteriosclerosis and the consequent increase in pulse wave pressure contribute to accelerating the process of atherosclerosis [59] (Figure 2).

**Figure 3:**
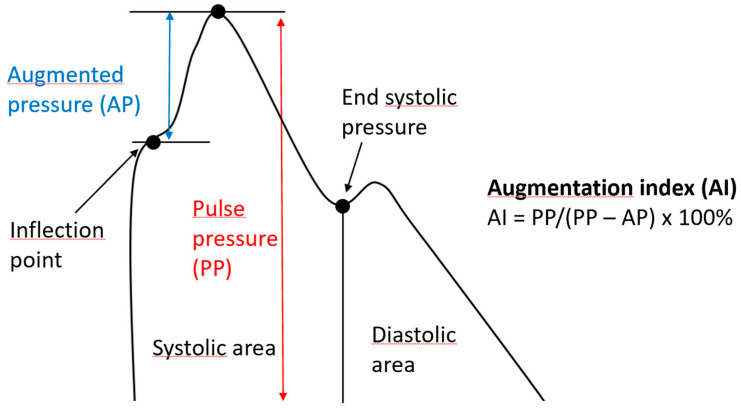
Graphical representation of the augmentation index. The augmentation index is expressed as a percentage and represents the ratio between pulse pressure (the difference between systolic and diastolic pressure) and the augmented pressure (the difference between central systolic pressure and the pressure of the reflected wave). The increase in arterial stiffness is directly proportional to the pressure of the reflected wave, and as a consequence, to the augmentation index.

Arteriosclerosis and its consequences (both cardiovascular and renal disease) have recently been shown to be associated more with central blood pressure (cBP) than with brachial blood pressure (bBP). Augmentation index (AIx) is an indirect measure of cBP that can be estimated non-invasively by applanation tonometry and represents the increase in pressure that occurs after the systolic peak, caused by the wave reflected from peripheral blood vessels (Figure 3) [60,61,62,63].

Aortic stiffness has been evaluated in different pathological conditions: chronic kidney disease, aortic valvular diseases, congenital heart disease, bicuspid aortic valve (BAV), Marfan syndrome (MFS), and hypertrophic cardiomyopathy [64,65,66]. Arterial stiffness can contribute to HCTDs pathophysiology, and higher values of PWV may be related to a more aggressive disease. MFS patients, such as LDS patients, have greater arterial stiffness compared to healthy volunteers [67]. In patients with MFS, changes in aortic compliance have been shown to predict the progression of descending aorta dilatation (24). High values in PWV have been shown to be related to the risk of aneurysm formation in the general population and its progression [67,68,69]. However, cfPWV is likely invalid for accurate arterial stiffness assessment in patients with AAA owing to the apparent confounding effect of aortic size [70].

These data support the usefulness of the evaluation of aortic function in a broad spectrum of thoracic aorta diseases, giving some important information: the relative independence of functional alterations from dimensional variations, their persistence even after the correction of some haemodynamic problems (e.g., aortic coarctation), and their identification also in the family members of patients who do not carry the proband phenotype (e.g., BAV) [71]. It is still a matter of debate whether, in aortopathies that are genetically determined, the study of aortic function can provide prognostic information different from the evaluation of the dimensions alone.

Drugs Effect on Aortic Stiffness

The control of cardiovascular risk factors, including hypertension, could limit arterial stiffness development. Benetos et al. [72] demonstrated a favourable decrease in the cfPWV after Ramipril administration, both in the acute phase (3 h after the first dose) and in the chronic one (after 15 days). Other trials have shown similar findings with different drugs within the same class [73,74]. The mechanism is mainly related to the reduction of wave reflection, with consequent lowering of systolic blood pressure and reduction in LV adverse remodelling. Optimal blood pressure control is so important to avoid high PWV and AS [75]. However, the use of PWV measurement is not so practical and is not yet recommended in routine clinical practice [49].

The REASON study (Regression of Arterial Stiffness in a Controlled Double-Blind Study) compared the association between perindopril/indapamide (2/0.625 mg/day) versus atenolol (50 mg/day) alone for 12 months in hypertensive patients. At 1 year, blood pressure reduction was greater in the first group compared to the second one [76]. Higher cfPWV values are considered a marker of more resistant hypertension with lower treatment efficacy. Nebivolol is a selective beta-1 blocker, and compared with atenolol, has shown a better decrease in augmentation index thanks to a vasodilatory effect related to nitric oxide production [77]. Moreover, it is important to consider that heart rate control with beta-blockers has an impact on the relationship between AS and central aortic pressure: heart rate reduction causes an increase in LV filling pressure, enhancing ventricular-vascular coupling and aortic pulse augmentation [78].

The CAFE (Conduit Artery Function Evaluation) trial compared the impact of atenolol/thiazide vs. amlodipine/perindopril associations on derived central aortic pressures and hemodynamic status in 2073 participants with hypertension and at least 3 additional risk factors [71]. The second group had lower values of central aortic pressure, primarily due to beta-blockers effects on heart rate and stroke volume.

However, because of the small sample size of most of the studies, more RCTs are needed to evaluate the effects of antihypertensive medication on AS.

Some small, randomized studies with statins have shown a reduction in inflammation markers, but their role is controversial at the moment [45]. Finally, it seems that rosuvastatin could reduce 3-nitrotyrosine levels (a marker of oxidative stress) and, consequentially, aortic PWV. Interestingly, reduction of plasma cholesterol was the only independent predictor of reduced arterial stiffness in patients with primary hypercholesterolemia after rosuvastatin therapy [79].

Type 2 diabetes is related to arterial stiffness due to the accumulation of advanced glycation end products in the ECM [80,81,82]. DPP-4 (dipeptidyl peptidase-4), GLP-1 RA (glucagon-like peptide-1 receptor agonists), and SGLT2i (sodium-glucose cotransporter 2 inhibitor) have been shown to slightly decrease PWV. A combination therapy with SGLT2i and GLP-1 RA seems to confer greater endothelial protection. However, more RCTs are needed to confirm therapeutic efficacy [83,84,85].

Regarding aortic disease interventional treatment, PWV has been shown to possibly predict complications and possible sac growth/shrinkage after EVAR [86,87]. Surgical replacement of aortic tissue with a prosthesis has been reported to increase PWV and therefore the risk of long-term cardiovascular and cerebrovascular events [88]. Minimizing treatment and using postoperative beta-blockers may be the best choices to attenuate this effect [89].

## 5. Conclusions

The mechanisms behind aortic pathologies are multiple and complex. Arterial stiffness has been revealed to have a strong connection with aortopathies, and its evaluation through PWV can be of great use for an early assessment of both risk in predisposed patients and their response to therapy. More RCTs are needed to assess PWV’s clinical indication. However, its use could influence the approach to CV risk stratification and CV disease management.

## Figures and Tables

**Table 1:** Summary of hereditary conditions leading to aortic disease.

Pathology	Transmission	Mutated Gene; Protein and Its Function	Cardiovascular Clinical Features	Other Clinical Features
Marfan Syndrome type 1	AD	FBN-1; fibrillin-1, structural component of microfibrils of ECM	Aortic root and ascending aorta dilatation, aneurysm, and dissection. Other features can include mitral valve prolapse or mitral valve calcification	Ectopia lentis, skin and skeletal manifestation, pneumothorax
Marfan Syndrome type 2	AD	FBN-1; fibrillin-1. TGFBR 1 and 2; receptors in the TGF-β pathway that have a crucial role in ECM production and differentiation	Aortic root and ascending aorta dilatation, aneurysm, and dissection	Skeletal manifestation, but no ectopia lentis
MASS	AD	FBN-1; fibrillin-1	Mitral valve prolapse, aortic dilation without aneurysm formation	Nonspecific skin and skeletal marfanoid features, but no ectopia lentis
Loeys–Dietz Syndrome	AD	TGFBR 1 and 2	Arterial tortuosity with rapidly progressive aortic aneurysms	Hypertelorism, bifid/broad uvula, or cleft palate
Vascular Ehlers–Danlos syndromes	AD	COL3A1 (more frequent); type III collagen COL1A1; type I collagen	Arterial fragility with aneurysm development, dissection, and rupture. Possible cardiac valve involvement	Skin and skeletal features, organ fragility, extensive bruising, and pneumothorax
Osteogenesis imperfecta	AD	COL1A1 and 2; type I collagen	Aortic dilatation and dissection, cardiac valve regurgitation	Scarce skeletal development is associated with bone fragility and smaller stature, blue sclerae, and hyperextensible ligaments
Arterial tortuosity syndrome (ATS)	AR	SLC2A10; GLUT10, that is linked with the TGF-β pathway	Tortuosity of the aorta and mid-sized arteries, as well as focal stenosis of segments of the pulmonary arteries and/or aorta	Atypical skin and skeletal features
Non syndromic aneurysm/dissection	/	NOTCH1; Nocht1, is important in cardiovascular embryogenesis. ACTA2; actin, a cytoskeletal protein MYH11; myosin, a cytoskeletal protein FBN-1; fibrillin TGFBR 1 and 2	Aortic dilatation and/or aneurysm, bicuspid aortic valve	No systemic manifestation

Legend. AD, autosomal dominant; AR, autosomal recessive.

**Table 2:** Indices of local arterial stiffness.

Index	Definition	Formula
Volume compliance	Change in arterial volume relative to the change in arterial pressure (influenced by wall stiffness, arterial size, and wall thickness)	ΔV/ΔP (mL/mmHg)
Arterial compliance	Area change for a given pressure step at fixed vessel length, estimation of compliance based on cross-sectional (rather than volume) measurements.	ΔD/ΔP (cm/mmHg) or (cm^2^/mmHg)
Arterial distensibility	Fractional change in cross-sectional area relative to the change in arterial pressure	(ΔD/ΔP × D) (mmHg^−1^)
Pulse wave velocity	Speed at which the arterial pulse propagates in the arteries (usually carotid-femoral pulse wave velocity)	PWV = √1/pDC (m/s)
Augmentation Index	The difference between the second and first systolic peaks as a percentage of pulse pressure	

## Data Availability

Not applicable.
